# Autism and Religion

**DOI:** 10.3390/children10081417

**Published:** 2023-08-20

**Authors:** Szabolcs Kéri

**Affiliations:** 1 Department of Cognitive Science, Budapest University of Technology and Economics, 1111 Budapest, Hungary; keri.szabolcs@ttk.bme.hu; 2 National Institute of Mental Health, Neurology, and Neurosurgery, 1145 Budapest, Hungary; 3 Department of Physiology, Albert Szent-Györgyi Medical School, University of Szeged, 6720 Szeged, Hungary

**Keywords:** autism spectrum disorder, religion, theory of mind, belief, mirror neuron, cognition

## Abstract

The disease burden of autism spectrum disorder (ASD) is a definitive public health challenge. The quality of life of children with ASD depends on how the cultural environment fits their special needs, including religious and spiritual factors. Does ASD predict low religiosity, and if not, what is the significance for clinical care? To answer this question, we reviewed the literature on the cognitive models of ASD and religious beliefs. We found that the cognitive models of ASD and religious beliefs substantially overlap, which is particularly important from a developmental psychological perspective. These models include Theory of Mind and intentionality, the “broken mirror” hypothesis, central coherence, and the intense world theory. We dispute the assumption that individuals with ASD are inherently less religious and spiritual than the neurotypical population. Religiosity is possibly expressed differently in ASD with unique spiritual experiences and beliefs (“gifted, visionary, and truth-seeker”). In some circumstances, a religious background can be helpful for both children with ASD and their caregivers. These circumstances should not be neglected, and clinicians are encouraged to consider patients’ religious context, resources, and needs.

## 1. Introduction: The Convergence between the Cognitive Science of Autism and Religion May Help Understand the Struggles of Affected Families 

### 1.1. Are People with Autism Atheists? 

In the era of information technology, the increasing secularization in Europe and East America has raised the concept that autism is the next step in human evolution as a fundamental basis of biologically rooted atheism and intelligence [1,2,3]. Jesse Bering (2002) observed in autobiographical accounts of individuals with autism that God is regularly an abstract and mechanical concept instead of a personally available, human-like agent with aims, intentions, and social relatedness [4]. The theory of “atheist autism” comes from the notion that religion and spirituality require a cognitive faculty that seeks purposeful intentions, wishes, thoughts, and emotions in others’ minds, that is, to mentalize in a teleological framework [5,6,7,8]. This is precisely what individuals with autism cannot accomplish in light of classic interpretations [5,9,10].

According to the cognitive science of religion, the experience and belief of divine interventions, supernatural minds, and intelligent design are evolutionary byproducts of hyperactive mentalization (attribution of mental states to explain behavior), teleological reasoning (attribution of purpose and meaning to objects and events), and agency detection (supposing that things with intentions act in the world). These cognitive byproducts obscure the true nature of the world’s random, dynamic, self-organizing, adaptive, and emergent rationality and causality [11,12,13,14]. As emphasized by Atran and Norenzayan (2004), religious cognition is a bias of information processing and representation, serving our naïve and intuitive understanding of the mechanical world (folkmechanics), living organisms (folkbiology), and human behavior with an inner world of subjectivity (folkpsychology). Religious beliefs only minimally diverge from everyday concepts (minimal counterintuitiveness, e.g., incarnation, afterlife, and human-like beings with wings, called angels) to facilitate the imagination of a supernatural world. This creates a framework for transcendent meaning, depicts overall life purposes, and answers existential and moral questions (birth, death, good and bad, deception, and suffering) [15]. Atran and Norenzayan (2004) summarized the role of folkpsychology in religious cognition as follows: “A key feature of the supernatural agent concepts common to all religions is the triggering of an Innate Releasing Mechanism, or Agency Detector, whose proper domain encompasses animate objects relevant to hominid survival-such as predators, protectors and prey-but which actually extends to moving dots on computer screens, voices in wind, faces on clouds. Folkpsychology also crucially involves metarepresentation, which makes deception possible and threatens any social order; however, these same metacognitive capacities provide the hope and promise of open-ended solutions via representations of counterfactual supernatural worlds that cannot be logically or empirically verified or falsified” [15].

### 1.2. The Unique Religiosity and Spirituality of Autism

The impaired religious understanding hypothesis of autism postulates that developmental deficits during childhood in folkpsychology disrupt the intuitive understanding of religious representations about intentional agents with counterintuitive properties [9,16]. However, in contrast to the simplified view that religious and spiritual thoughts are minimal, mechanistic, or absent in “mind-blind autism”, comprehensive descriptions suggest that some individuals with autism experience a rich imaginary world with benevolent invisible minds (bodiless agents of gods, angels, and spirits). These individuals live with unusual sensory impressions that bring stability and emotional safety, even when the external world is fragmented, incoherent, and overwhelming [17,18]. Ingela Visuri concluded that the results “do not conform to popular expectations that autistic minds are less adapted to experience supernatural agents, and it is instead argued that imaginative, autistic individuals may embrace religious and fictive agents in a search for socially and emotionally comprehensible interaction” [17].

Practically, religious culture often challenges children with autism and their caregivers. Some individuals with autism may deeply engage with and find comfort in religious and spiritual rituals and traditions, while others may have limited interest or struggle with religious concepts due to their unique cognitive abilities. Individuals with autism often have sensory sensitivities, which can impact their experience of spiritual practices. Moreover, they tend to interpret religious texts and concepts more literally, which can affect their understanding of metaphorical or abstract religious teachings. This literal thinking style may lead to unique perspectives on religious beliefs and practices [19,20]. Religious settings often involve unspoken social norms, which can be challenging. However, religious communities can provide a sense of belonging, understanding, and support. Acceptance and inclusion within these communities can benefit those who may feel marginalized in other social contexts. Religion can serve as a coping mechanism, providing comfort and a structured framework to navigate life’s difficulties. Moreover, religious practices may help with emotional regulation and provide a sense of stability. Therefore, inclusive religious communities should try to accommodate the needs of individuals with autism by giving sensory-friendly environments, adapted rituals, and educational materials [21,22].

The present review aims to outline the specific aspects of religious cognition in individuals with autism, with a special reference to children and their families. Beyond its theoretical significance, it is essential for patients and their caregivers from different religious environments because this sociocultural context substantially impacts their everyday activity, development, educational opportunities, and quality of life [23]. Therefore, we discuss the cognitive models relevant to autism and religious beliefs. These models include Theory of Mind and intentionality, mirror neurons and imitation, central coherence, and the intense world hypotheses. We review the essential characteristics of these models, and the related developmental psychological findings and then discuss how they explain the phenomenology of autism and religion. Emphasis will be laid on the critical evaluation of the models, their practical relevance for children’s mental health, and future directions for research. We use cognitive models to integrate diverse and contradictory findings from experimental and developmental psychology, clinical sciences, social sciences, religious studies, and cultural anthropology. Before discussing the cognitive models, we briefly introduce the current concept of ASD.

## 2. Methods

We followed the principles of SANRA (Scale for the Assessment of Narrative Review Articles) in the preparation of our narrative review: (1) justification of the article’s importance for the readership, (2) statement of concrete aims and formulations of questions, (3) description of the literature search, (4) referencing, (5) scientific reasoning, and (6) appropriate presentation of data [24]. This approach has limitations in the present case because we aimed to integrate results from a wide range of research areas with different conceptual frameworks and methods (experimental psychology, developmental psychology, clinical sciences, social sciences, religious studies, and cultural anthropology), taking into account research spanning decades. We searched the following databases from 1980 up to June 2023: PsycINFO, Scopus, Web of Science (including Arts & Humanities), PubMed, Semantic Scholar, Social Science Citation Index (SSCI), ATLA Religion Database, and Religious and Theological Abstracts. The keywords included “autism”, “religion”, “spirituality”, “cognitive”, “Theory of Mind”, “mirror neuron”, and “central coherence”. The reference lists of the 500 most highly cited papers were manually screened to identify relevant articles. Referencing aimed to consider research articles from all appropriate scientific fields. The findings were synthesized and presented in the following order: (1) introduction into the concept of the autism spectrum, (2) the Theory of Mind, (3) the mirror neuron hypothesis, (4) the central coherence and intense world hypothesis, (5) the unique religious style in autism and its everyday implications, and (6) conclusions and practical recommendations.

## 3. The Current Concept of Autism Spectrum Disorder (ASD)

### 3.1. Diagnosis and Epidemiology

The diagnostic concept of ASD originates in the pioneering work of Leo Kanner (1943) and Hans Asperger (1944) during the Second World War [25]. Many experts now discredit Asperger’s work because of his active collaboration with the Nazi regime, eliminating “children who could not fit in with the *Volk*: the fascist ideal of a homogeneous Aryan people” [26]. Tragically, “autism emerged in a society that strove for the opposite of neurodiversity” [27].

Kanner adopted the concept of autism from Eugene Bleuler’s “four As” that characterized schizophrenia: autism, (loosened) association, (restricted) affect, and ambivalence [28]. In this sense, autism reflects the absorption of individuals with psychosis in their inner world, detached from external reality. Strikingly, only the third edition of the *Diagnostic and Statistical Manual of Mental Disorders* (DSM-III, 1980) included infantile autism instead of childhood schizophrenia [25]. Today, ASD is broadly considered a highly variable combination of traits and symptoms affecting social interactions, language, and communication. Individuals with ASD display restricted-stereotyped interests and behaviors, from severe dysfunctions to social awkwardness and eccentricity [29,30].

The ASD model is consistent with epidemiological and molecular biological results. In the 1960s, Kanner’s autism was a rare condition (4 per 10,000 persons, almost exclusively boys), whereas a current meta-analysis from 74 studies (2008–2021) with 30,212,757 participants revealed a prevalence range between 0.6% (Asia) and 1.7% (Australia) [31]. Another systematic review update confirmed that approximately 1% of children receive the diagnosis of ASD (male-to-female ratio: 4.2), and the prevalence has been increasing over time with a considerable variation across sociodemographic groups (range: 1.09/10,000 to 436.0/10,000) [32]. Recently, increasing attention has been paid to the rarely recognized female autism with atypical symptoms, including internalized anxiety and depression, camouflaging (mimicking peers’ social behavior), uncertainty in self-schemas, attention deficit, hyperactivity, impulsive-compulsive symptoms, and anorexia nervosa [33].

### 3.2. The Molecular Genetic Architecture of ASD Indicates Unique Neurodevelopment and Cognition

In addition to the variety of clinical phenomenology, the molecular and genetic architecture of ASD is also heterogeneous, including RNA metabolism, splicing/processing, DNA and chromatin binding, chromatin organization, and histone modifications [34]. The burden of rare neurodevelopmental mutations is high in patients with ASD and intellectual disability, and low in the broader autism phenotype where common autism risk alleles dominate. ASD exhibits overlapping and similar features and pathophysiological mechanisms with non-affective and affective psychotic disorders (schizophrenia, schizoaffective disorder, and bipolar disorder), with a particular reference to social and neurocognitive impairment, delayed development, neurological soft signs, motor abnormalities, and shared perinatal and intrauterine risk factors (e.g., hypoxia, viral infections, toxins, and metabolic disturbances). The neurodevelopmental impact, cognitive impairment, and the role of rare genetic variants (e.g., copy number variations, high-risk protein-disrupting mutations) are most pronounced in conditions with intellectual disability, followed by autism/attention-deficit hyperactivity disorder, schizophrenia, schizoaffective disorder, and bipolar disorder, respectively [35,36,37]. Interestingly, common, low-risk genetic variants linked to ASD may be positively selected during evolution because they induce neuronal changes with beneficial cognitive consequences in modern civilization (e.g., high systemizing ability, sensory-perceptual sensitivity, decreased emotional reactivity, and focused attention). Accordingly, ASD-associated variants showed gene-expression enrichments in the brain with gene ontologies such as nervous system development, synapse organization, and axon growth that may be associated with childhood intelligence and educational attainment [38,39]. 

If one intends to understand the puzzling relationship between ASD and religion, it is helpful to consider cognitive functions shaped by genetic, molecular, and neurodevelopmental factors. Strikingly, some of the putative cognitive mechanisms of ASD are also implicated in religion and spirituality [9].

## 4. Theory of Mind (ToM): A Putatively Key Mechanism for Autism and Religion

### 4.1. The ToM-Debate in Autism

ToM or mentalization refers to the ability to attribute mental states to oneself and others. We understand that other people have different beliefs, desires, and intentions from our inner world. A widespread brain network is implicated in ToM, including the medial prefrontal cortex, posterior cingulum, and the temporoparietal junction, which is potentially affected in ASD [40,41,42]. Several classic experimental and developmental psychology papers reported that children with ASD have impaired ToM and perform suboptimally on tasks requiring mental state attribution [10,43,44,45,46]. A recent meta-analysis examined the ToM difference between ASD and neurotypical individuals (3205 adults with ASD and 3675 controls). People with ASD performed worse on all ToM tasks. However, the performance of the ASD group was poorer on reading and complex scene comprehension tasks compared with self–other processing and perceptual scene comprehension [47]. Moreover, the relationship between ASD and ToM is complex. Not all individuals with ASD have ToM deficits, and the alterations manifest heterogeneously [48,49]. Happé (1994) found that some individuals with ASD pass false belief tasks, suggesting developed ToM abilities [50]. However, even those who passed the standard ToM tasks exhibited dysfunctions in explaining story characters’ nonliteral utterances, a more naturalistic condition for mental state attribution [50].

Hwang et al. (2007) proposed a ToM Continuum Model, which may explain the variability of abilities in ASD [51]. Some patients are unaware of mental states, while others successfully map first-order mental states like beliefs and desires. Patients close to neurotypical individuals on the ToM continuum are aware of higher-order mental states at the metacognitive level (thoughts about thoughts). It should be borne in mind that individuals with ASD may have substantially different subjective experiences during ToM tasks, which significantly impacts their needs in a social environment. Their ToM experiences are systematic, inferred from observable behaviors of others, emotionally less differentiated, and unrelated to the self-representations [51]. More critically, by reframing data from the past 40 years of ToM research, Deschrijver and Palmer (2020) claimed that it is essential to make a distinction between ToM (inferring others’ mental states) and mental conflict monitoring (detecting the match or mismatch between our own and others’ mental states) [52]. According to this model, ASD is characterized by a deficit of mental conflict monitoring instead of a missing mental representation ability (“mindblindness”) [52]. This paradigm shift has profound consequences on understanding other social cognitive domains, “including perspective taking, moral judgments, and understanding irony and humor” [52].

A recent literature review posited that there is no empirical evidence that people with ASD are uniquely impaired in ToM, and the ToM tasks do not account for autistic traits, social interaction, and empathy in real-life situations [53]. Gernsbacher and Yergeau (2019) concluded: “the claim that autistic people lack a theory of mind fails empirically; it fails in its specificity, universality, replicability, convergent validity, and predictive validity” [53]. The ToM model is insufficient to account for persistent challenges in real-world social interactions and restricted interests–repetitive behaviors [54,55]. Moreover, ToM covariates with other features of ASD related to language and communication with a shared developmental trajectory [56,57].

In summary, ToM deficits are common in ASD as measured by conventional tests, closely tied to language, communication, and social adaptation. However, individuals with ASD display a considerable variety in ToM depending on the tasks used. ToM abilities fall on a continuum, manifest differently, and do not fully explain the symptoms. Therefore, a more nuanced and interdisciplinary understanding of mental state attribution is needed in ASD.

### 4.2. ToM and Religion

The human ability to attribute mental states to others plays a crucial role in the cognitive science of religion. ToM allows humans to believe in supernatural agents, like gods, spirits, and angels, that have their mental states [11,58,59,60,61]. We attribute purposes and willed actions to these supernatural and bodiless minds, which are often counterintuitive (e.g., gods know everything). According to the most widely accepted path model of religious cognition, ToM is the first step, followed by body-mind dualism and teleology (the soul and the biological body are separable, and things have purposes), eventually leading to religious, paranormal, and life-purpose beliefs [62].

However, some scholars argue that cognitive scientists should be careful not to overstate the role of ToM [63]. While ToM may enable beliefs in supernatural agents, it does not necessarily cause those beliefs or determine their content. Cultural, social, and historical factors also shape religious concepts via rituals [64,65]. ToM allows humans to participate jointly in religious rituals, chant together, and achieve a shared sense of rhythmic coordination. Through these embodied spiritual experiences, one can attain an “anti-structural” state of consciousness that facilitates social bonding. Thus, ToM plays a role in religious belief and practice related to social synchronization during altered states of consciousness [66,67,68].

However, factors other than ToM should also be considered in shaping religious thought, including the human experience of meaning, emotional attunement, and absorption [69,70]. A cognitive approach that relies too heavily on ToM representing intentions and thoughts fails to fully account for the diversity of religious traditions, experiences, and the role of meaning in spiritual life. For example, emotional empathy and ToM should be distinguished: emotional empathy is positively related to religiosity, whereas “cold” ToM is unrelated or even negatively related to religiosity [71]. Moreover, recent evidence suggests that contrary to the ToM hypothesis, children’s understanding of divine minds is based on learned ontological knowledge, not social cognitive functions.

In summary, while ToM is crucial to understanding how humans can believe in and relate to supernatural agents, cognitive scientists of religion should recognize its limitations, as in the case of ASD. Religious thought emerges from a complex interplay of mental capacities and cultural factors. ToM alone does not determine or fully explain religious belief and practice, as it does not explain the complete profile of ASD [72].

### 4.3. ToM from a Developmental Perspective

Many studies explored how and when different aspects of ToM emerge. False belief understanding, the ability to recognize that others can have beliefs different from reality, develops between ages 3 and 5 [73,74]. Three-year-olds struggle with false belief tasks, while most 5-year-olds pass [75]. Children’s understanding of knowledge access, or how people come to know things, also improves with age in parallel with ToM. Young children are biased toward the causal quest rather than the epistemic quest, but 4- to 4.5-year-olds demonstrate a more nuanced understanding of knowledge access [76]. The ability of natural pedagogy [77]-sharing knowledge and developing teaching abilities- requires an understanding of others’ knowledge and learning needs. It develops between ages 3 and 6 years. Older children teach for longer, explain more rules, use more strategies, and recognize more errors [78,79]. Altogether, ToM emerges gradually in early childhood, with different aspects developing at different rates. False belief understanding, knowledge access, and teaching skills all improve between ages 3 and 6 years, with correlations across domains. While the development of ToM is complex, by age 5–6 years, most children achieve a sophisticated understanding of others’ mental states.

The development of ToM is delayed in children with ASD, which appears to be specific to understanding mental states and prosocial behavior. Children with ASD show milder impairments in relationship recognition or understanding the animate-inanimate distinction [44,80,81,82]. Some higher-functioning children with ASD do develop a first-order ToM (the ability to attribute beliefs to others) but struggle with the second-order theory of mind (the ability to attribute beliefs about beliefs), although it depends on task complexity [83,84]. Interventions targeting ToM development by teaching children with ASD to recognize emotions, understand different perspectives, and engage in pretend play somewhat improved social and communication skills, although the results are not straightforward [84,85,86].

Children develop an understanding of human and divine minds over time [87,88]. As their ToM improves, children better attribute mental states to God and reason about religious concepts [89]. However, children’s concepts of divine minds and supernatural concepts seem to depend more on what they are taught about God’s nature than on their ToM abilities [89,90]. While children’s thinking becomes more abstract as they age, their religious thinking develops concretely. Children start to understand religious ideas like God, prayer, and the afterlife between ages 5 to 9 and continue refining these concepts into their teens. As children’s minds and morality develop, their religious feelings and thinking become more sophisticated. For example, by ages 7 to 12, children from religious families start aligning their beliefs about preexistence with their parents’ and religious doctrine. However, children still rely heavily on their intuitions that they existed primarily as emotional beings before birth. Fully grasping the religious theory of divine agents, birth, death, and the afterlife takes significant time and effort for children [91,92,93].

Around ages 3 to 7 years, children start to distinguish between ordinary and extraordinary minds [87]. As their ToM improves, children better understand human and supernatural agents [94]. However, children’s concepts of God depend on what their parents and culture teach them, though younger children see God in more human-like terms [95]. Several cognitive tendencies provide a foundation for children to develop religious concepts, including detecting agency, understanding minds, and distinguishing animate from artificial objects. While culture and teaching powerfully shape the content of children’s religious beliefs, intuitive cognitive faculties influence how readily children can acquire religious ideas, from “magical thinking” to religious doctrines, values, and attitudes [96,97].

The development of religious ideas, feelings, and attitudes in children with ASD is poorly understood because of methodological difficulties in studying how they view spirituality. Special care must be taken to help them understand religious concepts and practices [98]. Swanson (2010) proposed the “experiential religion” approach to faith formation for children with ASD, incorporating their unique cognitive style and social, emotional, and communicational needs. It is based on an attentional focusing procedure on feelings and bodily sensations instead of mental states, language, and other higher-order cognitive skills [99].

## 5. The “Broken Mirror Theory” of Autism and Religion

### 5.1. Mirror Neurons: Functions and Development

Mirror neurons are a class of cells in several parts of the brain (e.g., premotor cortex, inferior parietal lobe, and somatosensory cortex) that are active when an individual performs a willed action or observes another person performing the same action (e.g., grasping a cup or watching one’s peer grasps the cup) [100,101,102]. Complementing the ToM system, these neurons are thought to be crucial for understanding others’ actions, imitating behaviors, and developing social skills. Although initial evidence suggested that mirror neurons are implicated in the emergence of intentions, imitation, empathy, and language comprehension, they are more likely associated with lower-level functions (e.g., discriminating between different types of grip but not the intention of the agent) [103].

While mirror neurons have been extensively studied in adults, less is known about their development in children [104]. Some evidence suggests that mirror neuron functions may be innate or develop very early in infancy. For example, newborn infants can imitate facial expressions, suggesting an early mirroring mechanism [104]. However, mirror neurons representing hand actions may develop later via sensorimotor experience [105]. The function of mirror neurons is shaped by interactions between genetic predispositions and environmental input during development and can change based on differences in environmental constraints [106]. While mirror neuron functions were once thought to have evolved specifically for social cognition, they may instead emerge from general learning processes. These cells do not encode goals or intentions as a predefined, evolutionally hardwired function. Instead, their diverse properties can be explained by the context-dependent nature of associative learning [103,106].

### 5.2. Can Mirror Neurons Highlight the Link between ASD and Religion?

The research on mirror neurons and ASD brought initial enthusiasm. It has been revealed that children with ASD lacked mirror neuron activity in the inferior frontal gyrus when imitating and observing emotional expressions. The mirror neuron impairment correlated with the severity of social symptoms, suggesting that these cells are involved in interpersonal difficulties in ASD [107]. Initial evidence suggested that mirror neurons are linked to imitation, empathy, and various other aspects of social cognition, and, therefore, their dysfunction can explain the core symptoms of ASD. However, subsequent research challenged the “broken mirror” theory of ASD, revealing no convincing evidence that mirror neurons are disrupted in ASD [108,109,110]. Instead, impaired top-down regulation from the prefrontal cortex serving complex social and goal-directed information may be implicated in ASD [111]. In addition, individuals with ASD display a disconnection in the “mimicry pathway” between frontal and temporal mirror neurons that allows automatic imitation of observed actions, whereas planning and emulation are less affected [111].

Can the mirror neuron system build a bridge between ASD and religion? Interestingly, some scholars focusing on the neurocognitive background of religious behavior paid attention not only to ASD but also to the mirror neuron system. The fundamental assumption was that social simulation processes play a role in spiritual belief formation by allowing people to empathize with and perceive intent in imagined supernatural agents. Therefore, the mirror neuron system might contribute to religious beliefs by causing people to perceive intent and experience empathy towards supernatural agents, even though they cannot directly observe them [112]. Participating in religious rituals also requires imitation and interpersonal synchronization. However, religious cognition is not limited to goal attribution, empathy, simulation, and emotion regulation. For example, doctrinal knowledge recruits the semantic network, whereas experiential aspects of religion and spirituality are linked to episodic memory and mental imagery [113]. Moreover, as we discussed, ASD is characterized by impaired top-down regulation of the mirror neuron system and disconnection in the “mimicry pathway” [111]. Planning and emulation are less affected, which may be critical in religious social cognition. More research is needed to fully understand how mirror neurons and simulation might contribute to religious belief to delineate its relationship with ASD.

## 6. Weak Central Coherence with an Intense World: Do Unusual Experiences Contribute to Religion and Spirituality in ASD?

### 6.1. Do Individuals with ASD Experience a Fragmented World with Intense Spots?

The weak central coherence theory proposes that individuals with ASD have a detail-focused cognitive style and difficulty perceiving the “big picture”. This theory suggests that children with ASD struggle to integrate information into meaningful wholes and to extract the overall meaning or gist. Instead, they tend to focus on details and have difficulty utilizing context [114,115]. For example, some individuals with ASD are less susceptible to visual illusions that rely on global processing [114]. They also underperform on tasks requiring context, like homograph pronunciation [116] and false belief tasks [117]. However, some studies found less convincing evidence for weak central coherence and suggested alternative explanations [118]. Hoy et al. (2004) reported that children with ASD performed similarly to controls on a visual illusion task [116], and Burnette et al. (2005) found no connection between weak central coherence and social-emotional difficulties [119].

A complementary approach to the central coherence hypothesis is the intense world theory. This theory proposes that ASD is caused by hyper-functioning of local neural microcircuits in the brain, leading to hyper-reactivity, hyper-plasticity, and hyper-perception. Autistic children have an “intense world syndrome” where sensory and emotional experiences are overwhelming and painful. The excessive neuronal processing causes autistic individuals to become trapped in a “small repertoire of secure behavioral routines” to escape the “painfully intense world” [120,121]. Initial empirical evidence yielded that autistic persons “experience overly strong perception, attention, and cognitive evaluation to repetitive expressions, particularly negative emotional expressions. This result supports the intense world theory more strongly than the mindblindness hypothesis” [122].

### 6.2. Do a Fragmented and Hyperintense World Lead to Unique Spiritual Experiences?

The hyper-perception of local details detached from the whole object or event and their powerful impressions can lead closer to the world of religion and spirituality in ASD. First, as discussed above, ToM deficits may result in impaired mapping of mental representations, impaired self-schemas, and worse symbolic interpretation of texts, which may impede a nuanced representation of religious symbols and doctrines in ASD. However, perceptual and emotional hypersensitivity disconnected from the global social context can easily induce out-of-the-ordinary sensory and affective experiences that can build the foundation of a visionary and heuristic spiritual world. The key is sensory hypersensitivity and weak priori cognitive schemes (decreased top-down control that maintain goal and context). As suggested by Narzisi and Muccio (2021): “these two characteristics should be considered as a sort of phenomenological a priori that, importantly, could predispose people with ASD towards a spiritual experience, not intended in its religious meaning, but as an attribute of consciousness that consists of being aware of and attentive to what is occurring in the present moment” [123]. However, research focusing on the religion–autism enigma in the light of central coherence and intense world models is in its infancy, and definitive empirical work is needed.

## 7. Autism and Religiosity Revisited

### 7.1. The Nature of Religiosity in ASD: Gifted, Visionary, and Truth-Seeker

McCauley et al. (2019, 2020) argued that all three elements of the classic hypotheses on decreased religiosity in ASD are not sufficiently supported by empirical evidence, especially when the broader cultural context is considered [9,124]:

1. Social cognition content bias hypothesis: natural ToM operations are central in religious representations of intentional agents with counterintuitive properties (e.g., ghosts passing the wall, angels that never die, gods present everywhere simultaneously), cognitive appeal, and their intuitive inferential potential.
2. Impaired religious understanding hypothesis: deficits in ToM in ASD substantially limit intuitive understanding and inferences from religious representations about intentional agents with counterintuitive properties.
3. Mindblind atheism hypothesis: limitations on intuitive religious understanding and inference in ASD decrease the probability that they will be religious and increase the likelihood that they will be atheists.

The hypothesis that ASD is associated with atheism and that a religious environment may be uniformly stressful for the patients is probably an oversimplification. Evidence from cultural anthropology suggests that autistic symptoms may be considered divine or spiritual gifts instead of abnormal behavior and may represent certain advantages in religious and spiritual settings. In Hinduism, for example, separation from the social world and family ties left behind are among the most precious values [125]. Preoccupations with religious objects and materials (e.g., systemizing and memorizing sacred texts, working on clay ikons) and rhythmic-stereotyped movements during rituals are critical elements in the immersion of religious and spiritual spheres. In her groundbreaking book on autism, Uta Frith [46] mentions the case of Brother Juniper, an associate of Saint Francis of Assisi, who probably had ASD. He preferred seesawing for hours instead of social participation, interpreted the Franciscan principles of poverty literally instead of a mentalistic-symbolic stance, and stripped naked with stereotypical movements. In Judaism, learning and following God’s commandments is of fundamental importance that may help ASD individuals bypass God’s person-like mental states to focus on the ritualistic-behavioral aspects of religion [126,127]. Indeed, novel ethnographic research suggests that individuals with ASD have a distinct and unique understanding of religious ideas and supernatural concepts with a high level of systemizing and conceptual architecture [19].

Three fundamental cognitive mechanisms may contribute to the unique nature of religiosity in ASD [17,18]. First, compared to interaction with human minds via ToM, autistic individuals may see communication with bodiless agents from religions and other transcendent cultural systems as less complex because there is no need to process non-verbal communication (e.g., reading facial emotions, intonation, expressive body posture, and gestures) and real-life social attunement. Second, the absorption into transcendent spheres via imagination can paradoxically facilitate social cognition in ASD. Because of sensory hypersensitivity and impaired social perception, the external world is perceived as fragmented and incoherent in contrast to imaginary worlds that are predictable, emotionally safe, and houses controllable and benevolent mental states. Third, people with ASD frequently report sensing presence, touching, and seeing visions without sensory input. These experiences can be endowed with meaning by invisible agents from religions or other alternative realities [17,18].

Individuals with ASD are “truth seekers” that might posit a particular religious attitude concentrating on lawful events, unchangeable patterns and taxonomies, and systematic regularities. This is what dogmatics and systematic theology are about. Baron-Cohen (2008) set out in detail the hypersystemizing nature of ASD: “This can explain their preference for systems that change in highly lawful or predictable ways; why they become disabled when faced with systems characterized by less lawful change; and their need for sameness or resistance to change. If truth is defined as lawful patterns in data then, according to the hypersystemizing theory, people with ASD are strongly driven to discover the truth” [128]. However, because of hypoactive agency detection (ToM), some patients may avoid supernaturalism and prefer logical beliefs instead of metaphors (literal-mindedness and discomfort with symbolic fluidity). “Truth-seeking” and the need for sameness and predictability may lead to rigid and doctrinaire religiosity [16,129], but even these patients appreciate when a religious community accepts them [130].

### 7.2. The Lived Religiosity in Families with ASD

The relationship between religion and ASD is multifaceted and has practical implications. Several studies found that autistic traits can negatively impact traditional religious experiences. Schaap-Jonker et al. (2013) found that adults and children with ASD had a more negative view of God with ruling and punishing features. The authors suggested that difficulties in the social-interpersonal domain impacted religious and spiritual attitudes, but the importance of religion modified this relationship. In those who reported that religion was important in their life, the image of God was more positive and supportive [131]. In a non-clinical sample, similar results appeared: autistic traits were associated with greater fear of God and less positive feelings, especially in Calvinists [132].

However, other studies found that religion can be the cornerstone of coping and resilience for families of children with ASD. Positive religious coping was associated with better outcomes, including spirituality and stress-related growth in parents of children with ASD [133,134,135]. Kavaliotis (2017) found that religion strengthened resilience in parents of children with ASD, though Christian parents could better meet their child’s needs, possibly because they attributed less importance to divine will relative to Muslim families [136].

Several studies examined how religious communities can better support individuals with ASD and their families. Sometimes, children with ASD are excluded from religious activities. For example, they are refused to receive communion because they did not understand the meaning behind this ritual [137]. Sullivan and Aramini (2019) found that acceptance and inclusion were the biggest concerns for parents of children with ASD in religious education [138]. Boles (2019) aimed to understand the experience of children with ASD in the Catholic church using interviews and surveys with church staff members and parents of children with ASD. The results revealed that routine and suffering were essential factors. Boles (2019) argued that a holistic understanding of religious experience is needed to support people with ASD [139].

Some studies examined how culture and religious background shape views on ASD. Jegatheesan et al. (2010) found that South Asian Muslim immigrant families understood raising a child with ASD in religious terms. They strongly contested expert views, which they saw as undermining their child’s development [140]. The parents emphasized their children’s innocence and purity as a religious value, a “gift from Allah”. One of the mothers interviewed in the Jegatheesan et al. (2010) study held the conviction of reincarnation and thought that her son was in his “last rebirth”. He had not committed any sins intentionally and, consequently, would attain salvation. Among some South Asian Muslims, children with ASD are viewed as “very high spiritual beings... because of the transmigration of their souls” [140]. Jewish ultraorthodox mothers in Israel interpret ASD from both biomedical and spiritual-religious points of view. Metaphysical and moral accounts, with a particular reference to the transmigration of souls and suffering, link God’s mysterious ways and biological mechanisms underlying ASD [141].

In Latino families, mothers often reported that their children were blessings, tests, or signs from God [142]. Finally, beyond world religions, it is essential to bear in mind how New Age ideas impact ASD in affected families. The beliefs explaining ASD are various, including karma, reincarnation, spirit possession, toxication, and extraterrestrial experiments on humans that can lead to harmful treatments (e.g., specific diets, refusing vaccinations, purgation, and exorcism) [143]. Swinton and Trevett (2009) concluded that empathy and cultural openness are needed to understand the complex relationship between religion, spirituality, and ASD. Many people with ASD and their families want to engage deeply with faith, although it can also be problematic [144].

## 8. Conclusions and Recommendations for Clinical Practice

In summary, while autistic traits may present challenges for religious experience, religion also plays a vital role in coping for many families of children with ASD. Communities need a holistic understanding of spiritual experience to support people with ASD and their families. Cultural and religious background also shape how ASD is understood and experienced, which is precious information for the clinician and other professionals caring for individuals with ASD.

As we have seen, models explaining religious experiences and beliefs are parallel with cognitive models of autism, although the results are contradictory and do not explain ASD entirely. The classic emergence of intentional supernatural agents is generally weaker in patients due to impaired mental state attribution (ToM) and social synchronization. The understanding of the global context of religion is also less developed. Conversely, intensive sensory experiences, a tendency to systematization, and special subskills can produce a unique religious style in ASD.

When considering religious factors in the care of children with autism, it is essential to approach the situation with sensitivity, respect, and an open mind. The clinician should recognize and respect the diversity of religious beliefs and practices among families of children with ASD. Each family may have unique religious values and customs that influence their approach to healthcare and treatment. Open and transparent communication with the child’s parents and caregivers is crucial. The practitioner benefits from discussing religious beliefs and practices related to health and therapy to understand their preferences and any potential conflicts with standard medical interventions. We recommend creating an inclusive healthcare environment that respects and accommodates various religious practices, such as prayer times, dietary restrictions, or dress codes. Support families in advocating for their child’s needs within their religious community and provide resources to empower them in making informed decisions about the child’s care.

The treatment is more optimal when working collaboratively with the child’s family to develop a plan that aligns with their religious beliefs while also addressing the child’s specific needs. We must always be mindful of the confidentiality of faith-related information shared by the family and avoid imposing religious beliefs or judgments on them.

Some religious practices involve sensory experiences that might be challenging for children with autism, or they may report unusual experiences that can be falsely alarming for the clinician. The community must be aware of sensory sensitivities and adapt religious practices, if necessary, to make the child more comfortable.

Healthcare professionals should be educated about various religious beliefs and practices to understand their potential impact on the child’s care and be culturally competent. It is essential to embrace a multi-disciplinary approach to the child’s care, involving professionals who understand the cultural and religious aspects of the family’s beliefs. By considering and respecting religious factors, we can create a more inclusive and supportive environment for children with ASD and their families, fostering better outcomes for the child’s overall well-being.

## Data Availability

The paper contains no newly generated data.
